# Gen-Doping: aktuelle Nachweisverfahren und analytische Herausforderungen

**DOI:** 10.1007/s00103-026-04257-z

**Published:** 2026-06-03

**Authors:** Mario Thevis, Andreas Thomas, Nana Naumann

**Affiliations:** 1 Zentrum für Präventive Dopingforschung, Institut für Biochemie, Deutsche Sporthochschule Köln, Am Sportpark Müngersdorf 6, 50933 Köln, Deutschland; 2 European Monitoring Center for Emerging Doping Agents (EuMoCEDA), Köln/Bonn, Deutschland

**Keywords:** Massenspektrometrie, Leistungssteigerung, Transgen, Gen-Therapie, Sport, Mass spectrometry, Performance enhancement, Transgene, Gene therapy, Sports

## Abstract

Unter Gen-Doping versteht man den Einsatz gentherapeutischer Substanzen oder Technologien zum Zwecke einer unerlaubten Leistungssteigerung im Sport. Unterschiedlichste dopinganalytische Nachweisverfahren befinden sich aktuell in der Entwicklung und reichen von PCR (Polymerase Chain Reaction)-basierten Methoden über hochauflösende Massenspektrometrie bis hin zu auf CRISPR/Cas (Clustered Regularly Interspaced Short Palindromic Repeat/CRISPR-associated) basierenden Detektionsverfahren. Aufgrund der vielfältigen Ansätze in der gentherapeutischen klinischen Forschung ist davon auszugehen, dass auch verschiedenste Verfahren zum Einsatz kommen werden, um Gen-Doping-Fälle nachweisen zu können. Das zunehmende Angebot an nicht zugelassenen Gen-Doping-relevanten Produkten auf dem freien Markt unterstreicht die dringende Notwendigkeit, neue Nachweisverfahren in der Doping-Routine-Analytik zu etablieren.

## Abstract (en)

Gene doping refers to the use of gene therapeutic substances or technologies for the purpose of illicit sport performance enhancement. A wide variety of doping analytical detection methods are currently under development, ranging from PCR (Polymerase Chain Reaction) to high-resolution mass spectrometry to clustered regularly interspaced short palindromic repeat (CRISPR) and CRISPR-associated (CRISPR/Cas)-based detection methods. Due to the diverse approaches in gene therapy clinical research, it can be assumed that a wide variety of methods will also be needed to detect cases of gene doping. The increasing availability of unapproved gene doping-related products on the open consumer market underscores the urgent need to establish new detection methods for routine doping control analysis.

## Einleitung

Unter „Gen-Therapie“ versteht man die Einbringung von genetischem Material (direkt oder über genveränderte Zellen) in einen Patienten oder eine Patientin zur Milderung oder sogar Heilung einer Erkrankung und ihrer Symptome [1]. Gen-Doping beschreibt hingegen den missbräuchlichen Einsatz eben solcher Gentherapeutika im Sport, um im Wettkampf einen unerlaubten Leistungsvorteil zu erzielen.

Seit Beginn erster gentherapeutischer klinischer Studien in den 1990er-Jahren hat der Umfang an präklinischer Forschung und klinischen Studien rasant zugenommen und immer mehr Therapieverfahren finden Eingang in den klinischen Markt (*Gin et al.* berichten in ihrer Übersichtsstudie von über 3900 laufenden klinischen Studien in 46 Ländern und *Wang et al.* listen in ihrer Übersichtsarbeit insgesamt 43 zugelassene Therapieverfahren auf; [2, 3]). Untersuchte Gen-Therapieverfahren weisen mittlerweile ein sehr breites Anwendungsspektrum auf und reichen von Krebstherapien, die immer noch von einem Großteil der klinischen Studien adressiert werden, über die eher „klassischen“ Behandlungsfelder der Gen-Therapie, den monogenetischen Erkrankungen (wie z. B. muskuläre Dystrophien) bis hin zu eher neueren Anwendungsfeldern wie der Behandlung von Infektionskrankheiten (z. B. mit dem Hepatitis-B-Virus (HBV) oder dem humanen Immundefizienz-Virus (HIV); [2]). Gen-Therapieansätze umfassen zudem ein sehr breites Methodenspektrum. Sie werden entweder durch die direkte Einbringung einer gentherapeutischen Substanz in den Patienten oder die Patientin (*in vivo*) angewendet oder durch die genetische Veränderung von patienteneigenem Zellmaterial, welches im Anschluss dem Patienten oder der Patientin wieder zugeführt wird (*ex vivo*; [1]). Bei dem Transfer künstlicher Gene (sogenannter Transgene) werden synthetische Nukleinsäuren über zumeist virale oder bakterielle Trägersysteme (sogenannte Vektoren) in den Patienten oder die Patientin eingebracht, um z. B. ein körpereigenes, defektes Gen zu substituieren. Inhibitorische Therapieverfahren erfolgen beispielsweise über kurze, artifizielle Ribonukleinsäuren (RNAs), u. a. über sogenannte Silencer RNAs (Small Interfering RNA, siRNA), oder Antisense Oligonukleotide (ASOs), welche die Umschreibung einer RNA in ein fehlerhaftes Protein unterdrücken können und z. B. als hybride Lipid-Nukleinsäure-Nanopartikel (LNPs) in den Patienten oder die Patientin eingebracht werden [4]. Gen-Editierungsverfahren, z. B. über CRISPR/Cas-Systeme, in der Öffentlichkeit besser als „Genschere“ bekannt, gewinnen zudem zunehmend an Bedeutung [5].

Der Einsatz von Gen-Doping als leistungssteigernde Maßnahme wurde offiziell erstmals 2001 durch die „Gene Therapy Working Group“ des Internationalen Olympischen Komitees diskutiert und als Risiko für einen fairen Wettkampf im Leistungssport identifiziert [6]. Gen-Doping wurde darauffolgend im Jahr 2003 zum ersten Mal durch die Welt-Anti-Doping-Organisation (World Anti-Doping Agency, WADA) offiziell als verbotene Methode (M3) in die Verbotsliste aufgenommen [7]. Ein mögliches Anwendungsszenario für Gen-Doping im Leistungssport ist die *In-vivo-*Einbringung artifizieller DNA-Sequenzen mit dem Ziel, Kraft und Ausdauer zu steigern sowie die Regenerationsfähigkeit eines Athleten oder einer Athletin zu verbessern. Als relevante Transgene, welche über einen solchen Ansatz appliziert werden könnten, sind hier beispielsweise Erythropoietin (*EPO*) und vaskulärer endothelialer Wachstumsfaktor (*VEGF*) für eine erhöhte Oxygenierung der Muskulatur sowie Follistatin (*FST*), Wachstumshormon (*GH*), insulinähnlicher Wachstumsfaktor I (*IGF‑1*) oder Myostatin-(*MSTN-*)Inhibitoren für eine Induktion des Muskelwachstums zu nennen [8]. Relevante gentherapeutische Ansätze, beispielsweise zur Behandlung neuromuskulärer Erkrankungen mit Follistatin oder zur Behandlung kardiovaskulärer Erkrankungen über VEGF, befinden sich bereits in klinischen Studien [3, 9, 10]. Zudem werden bereits unkontrollierte Gen-Therapien mit im europäischen und amerikanischen Markt nicht zugelassenen Präparaten für transgenes Follistatin sowie VEGF angeboten und *EPO*-Desoxyribonukleinsäuren-(DNA-)haltige Präparate für eine Selbstapplikation frei zugänglich über internetbasierte Plattformen für den Sportmarkt beworben und vertrieben [11]. Obwohl bislang kein Fall von Gen-Doping nachgewiesen werden konnte, gibt es leider bereits Hinweise darauf, dass ein Interesse am unerlaubten Einsatz dieser Verfahren bzw. Substanzen im Leistungssport besteht [12], verbunden mit einem kaum kalkulierbaren Gesundheitsrisiko für die betroffenen Sportlerinnen und Sportler.

Trotz umfangreicher Ansätze zur Methodenentwicklung seit Aufnahme des Begriffs „Gen-Doping“ als verbotene Methode durch die WADA, dauerte es jedoch noch einige Jahre bis erste laboranalytische Nachweisverfahren ihren Einsatz in der Praxis gefunden haben. Als erste analytische Methode zum Nachweis von Gen-Doping wurde die Detektion von siRNAs durch hochauflösende Massenspektrometrie (MS) gekoppelt an Hochleistungsflüssigkeitschromatographie (Liquid Chromatography – High Resolution/High Accuracy Mass Spectrometry, LC-HRMS) durch die WADA im Jahr 2015 akkreditiert [13]. Im Jahr 2020 erfolgte dann die Akkreditierung einer quantitativen Echtzeit-Polymerase-Kettenreaktion-(*Realtime-*Quantitative-Polymerase-Chain-Reaction-, qPCR-)basierten Methode zum Nachweis künstlicher *EPO*-DNA [14]. Vielfache Ansätze befinden sich zudem in der Entwicklung und reichen vom Nachweis einzelner Transgene bis hin zu genomweiten Sequenzierungsansätzen. Der vorliegende Übersichtsartikel soll einen Überblick über die bisher entwickelten Verfahren und künftige zu erwartenden Entwicklungen bieten.

## Nachweis körperfremder Desoxyribonukleinsäuren

**Abb. 1:**
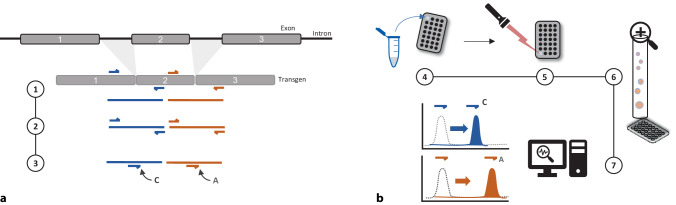
Übersicht zur Multiplex-Analytik mittels Polymerase-Kettenreaktion-(PCR-)gekoppelter MALDI-TOF-MS-(TOF-MS-Matrix-Assisted-Laser-Desorption/Ionization-Time-of-Flight-Mass-Spectrometry‑)Analyse. **a** Intronlose Transgene können über artifizielle Sequenzabschnitte an Exon-Exon-Grenzen (*EEG*) detektiert werden. Im ersten Schritt (1) findet eine Vervielfältigung der transgenen DNA über eine 20-plex PCR-Reaktion an unterschiedlichen EEGs statt. Um die Sensitivität der Methode zu erhöhen, wird im Anschluss eine zweite PCR-Reaktion auf denselben Sequenzbereichen durchgeführt (2). Kurze Extensionsprimer (*EP*) können in einem dritten Schritt (3) an die EEG-Sequenzabschnitte auf den PCR-Amplifikaten binden. Im Falle einer Bindung werden die EPs um ein einzelnes Nukleotid verlängert (komplementär zum Bindeabschnitt). **b** Für die MALDI-TOF-MS-Analyse werden minimale Mengen der finalen Extensionsreaktion auf einen mit einer Kristallisationsmatrix beschichteten Chip transferiert (4). Im Anschluss erfolgt eine Ionisierung der DNA-Moleküle mithilfe eines UV-Lasers (Wellenlänge = 337 nm; 5). Die so entstehenden, in der Regel einfach-positiv geladenen Moleküle werden in einem elektrischen Feld innerhalb eines Flugrohrs beschleunigt (6) und können nun in Abhängigkeit ihrer Masse und Ladung (*m/z*) und der damit verbunden „Flugzeit“ aufgetrennt und detektiert werden (7). Da Sequenz und Masse der EPs sowie der einzelnen Nukleotide, welche zu einer Extension eingesetzt werden, bekannt sind, kann über die MALDI-TOF-MS-Analyse über eine Masseverschiebung eine Auswertung erfolgen. Auf diese Weise können über die Methode insgesamt 7 unterschiedliche Transgene (*EPO, FST, IGF1, GH1, VEGFA, VEGFD, MSTN*) mit mindestens 2 Assays pro Transgen in einem einzigen Reaktionsansatz mit einer Sensitivität von bis zu 1500 Kopien pro mL nachgewiesen werden

Nach Bekanntwerden erster präklinischer Untersuchungsergebnisse für ein neues Gen-Therapeutikum („Repoxygen“, Oxford BioMedica) zur Expression von transgenem Erythropoietin für die Behandlung von Anämien im Jahr 2002 und Hinweisen auf Bestrebungen für einen missbräuchlichen Einsatz des Präparates im Leistungssport [15] konzentrierten sich erste Nachweisverfahren vor allem auf die Detektion von transgenem *EPO* [16, 17]. Im Jahr 2008 publizierten Beiter *et al.* einen ersten Ansatz für einen Nachweis von Transgenen (hier: *EPO, VEGF*), welche zum Zweck des Gen-Dopings eingesetzt werden könnten [18]. Die Strategie basierte auf der Hypothese, dass in der Gen-Therapie eingesetzte DNA-Konstrukte keine nichtkodierenden Sequenzinformationen (Introns) enthalten und somit künstliche, kodierende DNA-Sequenzübergänge (Exon-Exon-Grenzen, EEG) entstehen, die über PCR-Verfahren nachgewiesen werden können. Basierend auf dieser grundsätzlichen Strategie wurde in den folgenden Jahren eine Vielzahl von PCR-Nachweisverfahren, u. a. gegen humanes transgenes *EPO, VEGF, FST, IGF‑I* und *GH1*, entwickelt. Diese reichten von Standard-PCR-Verfahren [19, 20] über quantitative qPCR-Verfahren [21–29] bis hin zu Nachweismethoden mittels digitaler PCR (dPCR; [30, 31]). Es dauerte allerdings bis 2020, bis schließlich das erste Gen-Doping-Testverfahren für einen qPCR-basierten Nachweis von humaner, transgener *EPO*-DNA durch die WADA akkreditiert wurde [14]. Über 2 unterschiedliche Detektionsassays (zur Auffindung und Bestätigungsanalyse) kann hier eine Sensitivität von unter 500 Kopien pro Milliliter (mL) in humanem Vollblut oder 10 Kopien transgener DNA pro PCR-Ansatz erzielt werden. Aktuelle Entwicklungen konzentrieren sich zunehmend auf Multiplex-Strategien zum Nachweis verschiedener Transgene in einer Analyse. Beispielsweise bietet eine PCR-basierte Multiplex-Amplifikation gekoppelt mit anschließender MALDI-TOF-(Matrix-Assisted-Laser-Desorption/Ionization-Time-of-Flight‑)Massenspektrometrie die Möglichkeit eines Nachweises von mehreren Transgenen bzw. Exon-Exon-Übergängen in einer Reaktion (Abb. 1; [32]). Eine alternative multiplexe MS-Strategie kombiniert eine EEG-gerichtete PCR unter Verwendung von Desoxyuridin-Triphosphaten (dUTPs) mit einer anschließenden Hydrolyse und einem enzymatischen Verdau der Uracil-haltigen PCR-Amplifikate und einer LC-HRMS/MS-Detektion der Spaltprodukte zum simultanen Nachweis verschiedener equiner Transgene (*eGH1, eGHRH, eIL10;* [33]). Durch Vereinigung der PCR-Ansätze und Konzentrierung zwischen den einzelnen Reaktionsschritten kann eine Sensitivität von bis zu 25 Kopien pro mL in equinem Plasma erzielt werden. Zudem werden sowohl gerichtete als auch ungerichtete *Next-Generation-Sequencing-*(NGS-)Ansätze diskutiert [34, 35], da Befürchtungen bestehen, dass wildtypische Gen-Sequenzen durch Codon-Optimierung [36] oder gezielte Veränderung von Detektionssequenzen manipuliert werden könnten und so ein Nachweis umgangen werden könnte. Gezielte NGS-Ansätze zeigen jedoch bisher eine reduzierte Sensitivität gegenüber qPCR-basierten Verfahren (1296 Kopien vs. 11–14 Kopien transgener DNA in 1 µg genomischem DNA-Hintergrund [21, 35]) und eine Anwendung ungerichteter Ansätze scheint vor allem aus ethischer Sicht und Gründen des Datenschutzes in humananalytischen Verfahren eher unwahrscheinlich.

CRISPR/Cas-Systeme können nicht nur als gentherapeutisches Werkzeug eingesetzt werden, sondern eignen sich auch zum Einsatz in der molekularen Diagnostik [37]. Auch in der Dopinganalytik befinden sich Verfahren zur Detektion von Gen-Doping über Gen-Editierungssysteme in der Entwicklung. Yan *et al*. haben ein Nachweisverfahren vorgeschlagen, bei dem nach isothermer PCR-Amplifikation (d. h. einer DNA-Amplifikation, welche unter stabilen Temperaturen stattfindet) ein anschließender CRISPR/Cas12a-basierter Nachweis von transgenem *EPO, IGF‑1,* und *GH1* in humanem Plasma erfolgen kann [38]. Zur Detektion werden EEG-spezifische crRNAs (CRISPR-RNAs) eingesetzt, welche auf den über eine RPA-(Recombinase-Polymerase-Amplification‑)Reaktion amplifizierten Transgenen binden und im Anschluss über Cas12a-Enzyme gespalten werden können. Durch die Unterbrechung einer Förster-Resonanzenergietransfer-(FRET-)basierten Reaktion kann dann entweder ein Nachweis über ein sich veränderndes Fluoreszenzsignal mittels Fluoreszenz-Spektrophotometrie erfolgen oder über eine Streptavidin-Biotin-basierte Antikörperreaktion mittels Auslesung lateraler Flussteststreifen. Yi *et al.* haben ein CRISPR/dCas9-(deadCas9-)basiertes Verfahren zum Nachweis von transgenem *EPO *in humanem Vollblut entwickelt, bei dem eine Isolation der transgenen DNA über eine EEG-spezifische sgRNA (Single Guide RNA) durchgeführt wird, welche an FLAG-markierte dCas9-/Anti-FLAG-Antikörper-markierte magnetische Nanopartikel binden kann [39]. Über eine weitere sgRNA, welche sequenzspezifisch eine andere Exon-Exon-Grenze bindet und mit Biotin-markierter dCas9 gekoppelt ist, kann im Anschluss über eine kolorimetrische Streptavidin-Poly-Horseradishperoxidase-(HRP-)Reaktion ein Transgen-Nachweis erbracht werden.

Obwohl neben intravenösen vor allem intramuskuläre Injektionen ein wahrscheinliches Applikationsszenarium darstellen, stehen Muskelbiopsien nicht zu dopinganalytischen Zwecken zur Verfügung. In *In-vivo-*Studien in Affen und Pferden mit transgenem *EPO* konnte über PCR-basierte Verfahren ein Transgen-Nachweis von mehreren Wochen in Serum bzw. Plasma und im Vollblut sogar über mehrere Monate erbracht werden [26, 40, 41]. Als primäre Detektionsmatrix von Transgenen wird daher vorrangig Vollblut genutzt, da von einer Anreicherung von transgener DNA in peripheren mononukleären Blutzellen (Peripheral Blood Mononuclear Cells, PBMCs) ausgegangen wird (z. B. in Form von episomaler DNA nach Transduktion mit rekombinantem AAV (Adeno-Associated Virus); [40]).

## Nachweis körperfremder Ribonukleinsäuren

**Abb. 2:**
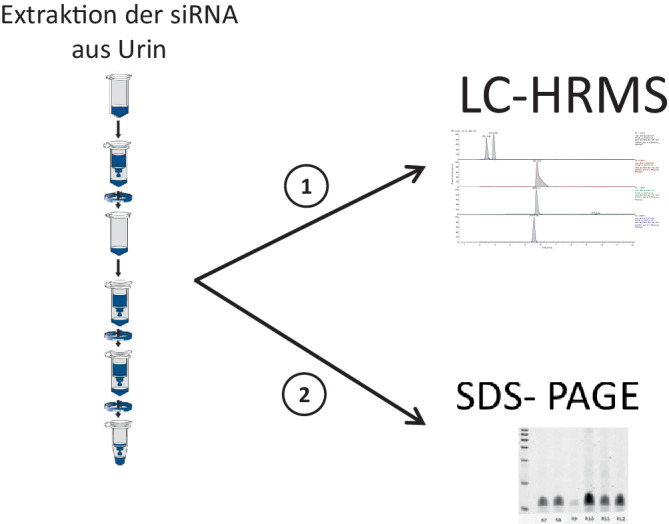
Nachweis von siRNA mittels LC-HRMS (Liquid Chromatography – High Resolution/High Accuracy Mass Spectrometry; 1) bzw. SDS-PAGE (Sodium Dodecyl Sulfate Polyacrylamide Gel Electrophoresis; 2) zu Dopingkontrollzwecken. Artifizielle siRNAs werden aus der Urinprobe aufgereinigt und nach alkaliner Hydrolyse mittels LC-HRMS oder gelbasiert mittels SDS-PAGE analysiert. Endogene RNAs werden durch die chemische Hydrolyse zersetzt. Chemische Modifikationen in exogenen siRNAs hemmen dagegen die Hydrolyse-Reaktion, sodass sie anschließend massenspektrometrisch bzw. gelelektrophoretisch erfasst werden können

Auch der missbräuchliche Einsatz von siRNAs zu Dopingzwecken ist prinzipiell denkbar. Hierbei wird der als RNA Interference (RNAi) bekannte biologische Abwehrmechanismus der Zelle genutzt, um gezielt Gene stillzulegen (*Knock-down*). Mithilfe von frei designbarer siRNA kann sequenzgesteuert gezielt Messenger RNA (mRNA) gebunden und anschließend abgebaut werden. Die Expression von bestimmten Genen kann somit effektiv verhindert werden. Dieser Vorgang ist nicht permanent und nach Absetzen der siRNA-Zufuhr wird auch die Stilllegung des Gens wieder aufgehoben. Als Zielgen bietet sich hier beispielsweise das muskelregulierende Myostatin-Gen an, welches in der Vergangenheit auch schon erfolgreich durch die Gabe von siRNA in Mausmodellen beeinflusst wurde [42]. Eine solche Manipulation ist prinzipiell nachweisbar, weil die zur siRNA-Synthese verwendeten Nukleotide chemisch verändert sind, um damit die biologische Stabilität zu verbessern und den schnellen Abbau im Organismus zu verhindern. Die chemischen Modifikationen sind für alle Nukleinsäurebausteine gut etabliert und haben unterschiedliche Effekte auf die Stabilität und den Transport in den Zellkern. Auch ihr Einfluss auf die Immunaktivität ist gut untersucht. Es kann also grob eine Klassifizierung nach Modifikationsstelle vorgenommen werden (Phosphonat‑, Ribose- oder Basen-Modifikation). Wichtige Modifikationen sind beispielsweise Phosphothioate, 2′-O-Methyl, 2′-Deoxy-2′-Fluoro, 2′-Benzyl und Locked Nucleic Acid (LNA; [43]). Basierend auf dem Nachweis dieser eindeutig unnatürlichen Modifikationen mittels LC-HRMS und gelbasierten Methoden (Sodium Dodecyl Sulfate Polyacrylamide Gel Electrophoresis, SDS-PAGE) konnte der erste Gen-Dopingnachweis entwickelt werden, der in einem Anti-Dopinglabor akkreditiert wurde [13]. Hierbei zeigte sich, dass die gute Stabilität der eingesetzten siRNA (Anti-Myostatin Oligonukleotide) insbesondere auch den Nachweis in Urinproben ermöglicht, die im Dopingkontrollszenario die am weitesten verbreitete Matrix sind. Und auch der Einsatz der LC-HRMS und SDS-PAGE ist vorteilhaft, weil diese Methoden in allen Dopingkontrolllaboratorien etabliert sind und zur Verfügung stehen. Abb. 2 zeigt den schematischen Ablauf der Methode.

## Nachweis von Gen-Editierung

**Abb. 3:**
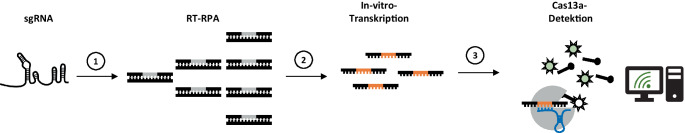
Nachweis von sgRNA (Single Guide RNA) über RT-RPA (Reverse Transcription-Recombinase Polymerase Amplification) und SHERLOCK (Specific High Sensitive Enzymatic Reporter UnLOCKing). Synthetische sgRNA-Moleküle werden zum Nachweis über das SHERLOCK-Verfahren erst in komplementäre DNA (cDNA) umgeschrieben und über eine isotherme PCR (RT-RPA) vervielfältigt (1). In der Cas13a-vermittelten SHERLOCK-Reaktion ist das Vorliegen einer RNA-Sequenz (*orange*) erforderlich, daher wird die cDNA mittels *In-vitro-*Transkription wieder über eine T7-RNA-Polymerase-vermittelte Reaktion in RNA umgeschrieben (2). Eine sgRNA-spezifische crRNA (CRISPR-RNA, *blau*) kann nun an die RNA-Sequenz binden und induziert hierdurch die Spaltung eines Reportermoleküls (*Stern*) über Cas13a (*grau*) und damit eine Fluoreszenzemission, welche über eine Fluoreszenzspektrophotometrie detektiert werden kann (3)

Gen-Editierungen bieten die Möglichkeit, präzise Veränderungen am Genom, und mittlerweile auch Transkriptom, eines Organismus vorzunehmen. Auf diese Weise kann eine defekte Gen-Sequenz beispielsweise „stillgelegt“ oder repariert werden. Eines der bekanntesten Werkzeuge der Gen-Editierung ist die CRISPR/Cas-Technologie, welche 2016 erstmals in klinischen Studien angewendet wurde [5]. Im Jahr 2023 ließ die FDA (Food and Drug Administration) erstmals eine Therapie zu, die eine Genomeditierungstechnologie für die *Ex-vivo-*Behandlung von Sichelzellanämie basierend auf dem CRISPR/Cas-Verfahren nutzt [44, 45]. Ein Verbot von Geneditierungsverfahren zur missbräuchlichen Anwendung im Leistungssport wurde durch die WADA im Jahr 2018 auf die Verbotsliste aufgenommen. Eine Anwendung von Geneditierungsverfahren zum Zwecke einer Leistungssteigerung ist im Pferdesport bereits Realität [46, 47]. Erste Nachweisverfahren wurden durch Paßreiter *et al.* entwickelt und richten sich gegen die bei CRISPR/Cas-Gen-Editierungen eingesetzte sgRNA [48, 49] sowie gegen das Cas9-Protein [50]. Zum Nachweis der synthetischen sgRNA wendet das Verfahren eine Amplifikation über Reverse Transkriptase-(RT-)RPA gekoppelt mit einer (ebenfalls CRISPR/Cas-basierten) SHERLOCK-(Specific-High-Sensitivity-Enzymatic-Reporter-UnLOCKing‑)Reaktion an (Abb. 3). Mit diesem Verfahren kann ein Nachweis von sgRNAs mit einer Sensitivität von bis zu 1 fM in 100 µL humanem Serum (äquivalent zu 3000 Kopien als Einsatz in die RT-RPA-Reaktion) erzielt werden.

Neben dem Nachweis der sgRNA aus dem CRISPR/Cas-Komplex kann auch der Nachweis des Proteinanteils (Cas9) zu Dopingkontrollzwecken herangezogen werden. Hierbei handelt es sich um ein DNA-schneidendes Enzym (Endonuklease oder „molekulare Schere“) mit bakteriellem Ursprung. Mithilfe von gut etablierten Methoden aus der Proteinanalytik (immobilisierte Antikörper und magnetische Partikel) kann dieses Enzym effektiv aus Blutproben isoliert, enzymatisch hydrolysiert und die charakteristischen Peptide (T25, T129 und T285) mittels LC-HRMS detektiert werden [50]. Diese Methodik wurde bereits erfolgreich *in vivo* getestet und zeigte hierbei, dass ein Nachweis des Cas9-Proteins nach einer einmaligen Applikation (bei Mäusen) für mehrere Stunden möglich war. Diese Methode kann somit als komplementär zur Detektion der sgRNA (siehe oben) verstanden werden und ermöglicht das Aufdecken von Dopingversuchen mit CRISPR/Cas unter Berücksichtigung des gesamten RNA-Protein-Komplexes.

Akiyama *et al.* schlagen ein Nachweisverfahren für CRISPR/Cas9-Ribonukleoprotein-(RNP-)Komplexe über RNA-Immunopräzipitation mittels Anti-Cas9-Antikörper-markierter magnetischer Nanopartikel, anschließender reverser Transkription (RT) der aufgereinigten sgRNA und sequenzspezifischer qPCR (RIP-qPCR) vor [51]. Es konnte eine Sensitivität von bis zu 0,01 ng/mL (294 fM) in humanem Plasma erzielt werden. Das Verfahren ermöglicht damit sowohl einen Nachweis der synthetischen sgRNA als auch die Ermittlung der für die Gen-Editierung vorgesehenen Zielsequenz, die jedoch sehr vielfältig sein kann. Ein spezifischer CRISPR/Cas-Nachweis ist daher immer noch eine der großen Herausforderungen in der Gen-Doping-Analytik.

## Fazit

Seit Beginn erster gentherapeutischer klinischer Studien sind die Technologien und Anwendungsfelder für Gen-Therapien rasant gewachsen. Nicht zugelassene Gen-Doping-Präparate und -Behandlungsverfahren werden bereits beworben und auf dem freien Markt angeboten und unterstreichen die Brisanz des Themas für die Anti-Doping-Analytik. Dopinganalytische Verfahren versuchen mit diesen Entwicklungen Schritt zu halten und reichen von Singleplex-PCR-Verfahren bis hin zu Hochmultiplex-massenspektrometrischer Analytik. Aufgrund der Vielzahl an möglichen Methoden und Zielsequenzen, welche im Fall eines Gen-Dopings zum Einsatz kommen könnten, ist davon auszugehen, dass es synergistischer Verfahren für einen Nachweis bedarf. Für die Dopinganalytik ergibt sich daraus die Notwendigkeit, Nachweisstrategien zu kombinieren. Besondere Herausforderungen bestehen in der Vielfalt potenzieller Zielsequenzen, der Wahl geeigneter Probenmatrices, den Nachweisfenstern sowie in der Gefahr von gezielten Sequenzmanipulationen. Künftige Entwicklungen sollten daher analytische Sensitivität, potenzielle Manipulationsstrategien, aber auch ethische und datenschutzrechtliche Anforderungen gemeinsam berücksichtigen.

## Data Availability

Alle dieser Arbeit zugrunde liegenden Daten sind in diesem Artikel enthalten.
